# Examining the Interaction of the Gut Microbiome with Host Metabolism and Cardiometabolic Health in Metabolic Syndrome

**DOI:** 10.3390/nu13124318

**Published:** 2021-11-29

**Authors:** Serena Galié, Christopher Papandreou, Pierre Arcelin, David Garcia, Antoni Palau-Galindo, Laia Gutiérrez-Tordera, Àlex Folch, Mònica Bulló

**Affiliations:** 1 Department of Biochemistry and Biotechnology, Faculty of Medicine and Health Sciences, University RoviraiVirgili (URV), 43201 Reus, Spain; serena.galie@outlook.it (S.G.); toni@comt.org (A.P.-G.); laia.gutierrez@estudiants.urv.cat (L.G.-T.); alexandre.folch@urv.cat (À.F.); 2 Institute of Health Pere Virgili—IISPV, University Hospital Sant Joan, 43202 Reus, Spain; parcelin@grupsagessa.com; 3 CIBER Fisiopatología de la Obesidad y Nutrición (CIBEROBN), Instituto de Salud Carlos III, 28029 Madrid, Spain; 4 Atención Basica de Salut (ABS) Reus V. Centre d’Assistència Primària Marià Fortuny, SAGESSA, 43204 Reus, Spain; 5 ABS Alt Camp Oest, Centre d’Atenció Primària, 43460 Alcover, Spain; dgarcia@absaco.org

**Keywords:** metabolites, gut microbiota, cross-talk, cardiovascular risk, metabolic syndrome, metabolism, obesity, microbial metabolites, omics, metabolomics

## Abstract

(1) Background: The microbiota-host cross-talk has been previously investigated, while its role in health is not yet clear. This study aimed to unravel the network of microbial-host interactions and correlate it with cardiometabolic risk factors. (2) Methods: A total of 47 adults with overweight/obesity and metabolic syndrome from the METADIET study were included in this cross-sectional analysis. Microbiota composition (151 genera) was assessed by 16S rRNA sequencing, fecal (m = 203) and plasma (m = 373) metabolites were profiled. An unsupervised sparse generalized canonical correlation analysis was used to construct a network of microbiota-metabolite interactions. A multi-omics score was derived for each cluster of the network and associated with cardiometabolic risk factors. (3) Results: Five multi-omics clusters were identified. Thirty-one fecal metabolites formed these clusters and were correlated with plasma sphingomyelins, lysophospholipids and medium to long-chain acylcarnitines. Seven genera from Ruminococcaceae and a member from the Desulfovibrionaceae family were correlated with fecal and plasma metabolites. Positive correlations were found between the multi-omics scores from two clusters with cholesterol and triglycerides levels. (4) Conclusions: We identified a correlated network between specific microbial genera and fecal/plasma metabolites in an adult population with metabolic syndrome, suggesting an interplay between gut microbiota and host lipid metabolism on cardiometabolic health.

## 1. Introduction

The metabolic variability derives from a complex and dynamic interaction between endogenous metabolism, environmental factors and the gut microbial ecosystem. Although gut microbiota is considered quite stable in adulthood, several modifiable factors can modulate its composition and activity. Whether these changes could compromise health is not yet well understood [1]. Therefore, a better understanding of how microbial communities affect or modify the complex environment of the human organism is a promising approach for the design of preventive and therapeutic strategies in different conditions [2]. In this sense, previous studies have suggested a role of some gut microbiota-derived metabolites, like bile acids and short-chain fatty acids (SCFAs), branched-chain amino acids (BCAAs), trimethylamine N-oxide, tryptophan and indole derivatives in the host-microbial cross-talk [3,4]. Furthermore, several fecal and circulating metabolites have been associated with clinical features associated with cardiometabolic risk [5], but it remains difficult to determine whether they are fully microbiota-derived or if other sources, including diet or the host itself, are also involved. An integration of metagenomics and metabolomics information may advance our knowledge on microbiota-host interactions [6]; however, to date, limited multi-omics analyses have been conducted. Two recent studies suggested a microbiota-host cross-talk analyzing the correlations of fecal and blood metabolites with gut microbiota composition by using both 16S rRNA and whole metagenomic shotgun sequencing in a large sample of UK adults (TwinsUK) [7,8]. However, whether a multi-omics profile characterizing this cross-talk could be associated with cardiometabolic health is unknown. Metabolic syndrome (MetS), a cluster of cardiometabolic conditions, is often accompanied by an imbalance of the gut microbiota [9] and alterations in metabolic pathways [5]. Identifying a host-microbial cross-talk in a population with MetS and its relationship with MetS features could further advance the understanding of biochemical processes preceding the development of cardiometabolic diseases.

Therefore, the aim of this study was to decipher the network of correlations between microbial genera, fecal and plasma metabolites using a multi-omics integrative approach in adults with overweight/obesity and MetS. Furthermore, we examined whether the identified multi-omics profiles were associated with cardiometabolic risk factors.

## 2. Materials and Methods

This is a cross-sectional analysis nested within the METADIET study, a randomized, controlled, crossover, dietary-intervention trial conducted in 50 adults with overweight/obesity and MetS [10]. Community-dwelling adults aged 30–65 years, with a body mass index (BMI) of 25–34.9kg/m^2^ who met at least three of the five diagnostic criteria of MetS and who regularly consumed a non-MedDiet were included in the study. Subjects were excluded if they suffered from type 2 diabetes (T2D), chronic diseases, had secondary obesity or related pathologies, non-controlled hypertension, LDL-cholesterol > 160 mg/dL, triglycerides > 400 mg/dL, followed specific pharmacological treatments (anti-inflammatory, corticoids, hormones or antibiotics), were alcohol or drug abusers and consumed prebiotics, probiotics or laxatives. Written informed consent was obtained from all study participants. The Institutional Review Board approved the study protocol, which accomplishes the ethical standards of the Declaration of Helsinki.

Weight, height and waist circumference were determined with calibrated scales and a wall-fixed stadiometer, and BMI was calculated. Blood pressure was measured in duplicate using a validated semiautomatic oscillometer (Omron Electronics Iberia S.A.U., Madrid, Spain). Blood and fecal samples were collected in fasting conditions before any intervention. Glucose and lipid profiles were measured using standard enzymatic automated methods. LDL-cholesterol was estimated using the Friedewald formula in subjects with triglycerides < 400 mg/dL. Circulating insulin levels were measured by commercial ELISA (Deltaclon SL, Madrid, Spain). The homeostatic model assessment of insulin resistance (HOMA-IR) was estimated [11]. Participants were instructed to collect stool samples in hermetic sterile flasks and freeze them immediately at −20 °C. Frozen samples were delivered to the laboratory within 1–2 days after collection and stored at −80 °C.

The fecal metabolomics profiling included 226 metabolites derived from a dual analytical approach (Supplementary Table S1 [12]). Ninety-four metabolites were quantified by a targeted analysis, using nuclear magnetic resonance (NMR) and liquid chromatography coupled to triple quadrupole mass spectrometry (LC-qTOF), while 132 metabolites were analyzed and semi-quantified with an untargeted approach. Particularly for the targeted analysis, NMR-based metabolic profiling of 37 metabolites included SCFAs, alcohols and organic acids, while LC-qTOF was used for the determination of 16 bile acids and 41 amino acids. The analytical procedures are specified in the Supplemental methods of [12]. Plasma metabolites (m = 378) were analyzed by different analytical platforms (Supplementary Table S2 [12]). LC-QqQ was used to analyze TMAO and derivatives, acylcarnitines, amino acids and serotonin. LC-qTOF was used for the lipidomic analysis. Measurements of total fatty acids, together with organic acids and sugar metabolites, mainly belonging to the tricarboxylic acid cycle, were obtained by a GC-qTOF analytical platform. A further description of the analytical procedures can be found in the Supplemental methods of [12].

Fecal DNA extraction was performed using a QIAmpPowerFecal DNA kit (Qiagen, Germantown, TN, USA) with a previous 5-minute lysis step (FastPrep-24-5G Homogenizer, MP Biomedicals). The 16S rRNA gene was amplified (Ion Metagenomics kitTM (Life Technology, Carlsbad, CA, USA), performing two separated PCR reactions with two primer sets (to amplify different hypervariable regions of 16S rRNA: V2, V4, V8 and V3, V6–7, V9). Amplicons were processed to obtain DNA libraries (Ion Plus Fragment Library kit and Ion Xpress Barcodes Adapters, 1-64 (Life Technology, Carlsbad, CA, USA) and adapter-ligated and nick-repaired libraries were purified (CleanNGSkit, CleanNA, Waddinxveen, The Netherlands). The libraries were further amplified (Ion Plus Fragment Library kit, Life Technology, Carlsbad, CA, USA) and quantified with Bioanalyzer (Agilent DNA 7500 Reagents, Agilent Technologies, Santa Clara, CA, USA). Equimolar amounts of all the libraries (60 µM) were sequenced in 4 different runs with Ion 520 and Ion 530 Kit-Chef (Life Technologies, Carlsbad, CA, USA) in an S5 sequencer from the Ion Torrent platform. Fastq data from sequencing were pre-processed with an adapted in-house script [13] in order to split only forward reads of each sample data into 6 subsets of 6 hypervariable regions. Forward reads from the V4 region were used for this study. Quality control, length filtering at 280 bp, and denoising of sequences with DADA2 pipeline other than taxonomy assignment were performed in QIIME2 software package using the latest version of Silva 132 as 16S rRNA gene classifier database. Finally, a further filtering step of ASV (Amplicon Sequence Variant) table at 10% prevalence cut-off at the taxonomic level of genera was achieved in R (using phyloseq package functions).

Twenty-three fecal metabolites were removed from the analysis due to the high number of missing values (>20%), and a total of 203 metabolites were included. In those metabolites with less than 20%, missing values were imputed using the random forest imputation approach (“missForest” function of “randomForest” R package version 4.6-14). The concentrations of metabolites were normalized and scaled to multiples of 1 SD with the rank-based inverse normal transformation.

Five out of 378 plasma metabolites were removed because of the high number of missing values (>20%), and the remaining missing values were imputed using the same approach as above. The rank-based inverse normal transformation was used to normalize their concentrations.

Baseline values of absolute abundances of the ASV table at the taxonomic level of 151 genera were center-normalized with clr function from the “composition” package on R (version 1.4-40). Due to the high dimensionality and collinear nature of the data, a Sparse Generalized Canonical Correlation Analysis (SGCCA) in an unsupervised mode was conducted to select the most relevant variables from three omics datasets (fecal metabolites, plasma metabolites, 16S rRNA). The model was implemented by using the canonical mode of the “wrapper.sgcca” algorithm [14], available in the mixOmics package in R (version 6.14.0) (Available online: http://mixomics.org/ (accessed on 20 November 2021)). The tuning procedure to identify the optimal number of components for each omic dataset was performed by a separated performance analysis on both tri and dual-omics datasets by using the “perform” function, which is available for both “block.splsda” and spls models. We then validated the choice of the optimal parameters by evaluating the AVE values of our obtained model of spls models (perf function in mixOmics package in R, version 6.14.0). A network analysis was implemented by the network function with a cut-off correlation value of 0.6 [15]. The network was further analyzed in Cytoscape software (version 3.8.2) (Available online: https://cytoscape.org/ (accessed on 20 November 2021)) in order to better visualize the presence of connected components and the relevant associations between fecal and plasma metabolites and microbial genera. A prefuse force-directed layout with a color grouping visualization based on the nature of components was selected to better feature the clusters of the network. A prefuse force-directed layout in Cytoscape is based on the force-directed-layout algorithm, which uses repulsive forces between nodes and attractive forces between adjacent nodes. The multi-omics score was calculated based on the weighted sum of the selected components in each cluster. The weights were obtained from the scaling with the eigengene centrality scores of each component in each module measured with the Page Ranking algorithm in Cytoscape. Linear regression models were fitted to examine the association between the derived scores and cardiometabolic risk factors (glucose, insulin, HOMA-IR, total cholesterol, HDLc, LDLc, VLDLc, triglyceride levels, systolic and diastolic blood pressure) adjusting for age, sex and BMI values. Furthermore, a Pearson partial correlation analysis adjusting for age, sex and BMI, was implemented between individual components of each multi-omic score and the cardiometabolic parameters significantly correlated with the scores. Values of the selected metabolites in feces and plasma, as well as the selected genera, were correlated with the cardiometabolic parameters, using the “associate” function from the “microbiome” package in R (version 1.12.0). A Benjamini-Hochberg false discovery rate (FDR) approach [16] was used to correct *p*-values for multiple testing.

All analyses were performed using R, version 3.6.2. All tests were two-sided, and significance was defined as *p* < 0.05.

## 3. Results

Of the 50 participants initially included in the METADIET study, three were excluded because of the unavailability of either 16S rRNA sequencing data or fecal metabolomics, resulting in a final total number of 47 participants. The general characteristics of participants are shown in Table 1. The mean age of participants was 50.6 ± 7.13 years, and the mean BMI was 30.5 ± 2.28 Kg/m^2^.

### 3.1. Multi-Omics Network of Correlations between Gut Microbiota, Fecal and Plasma Metabolites

**Figure 1:**
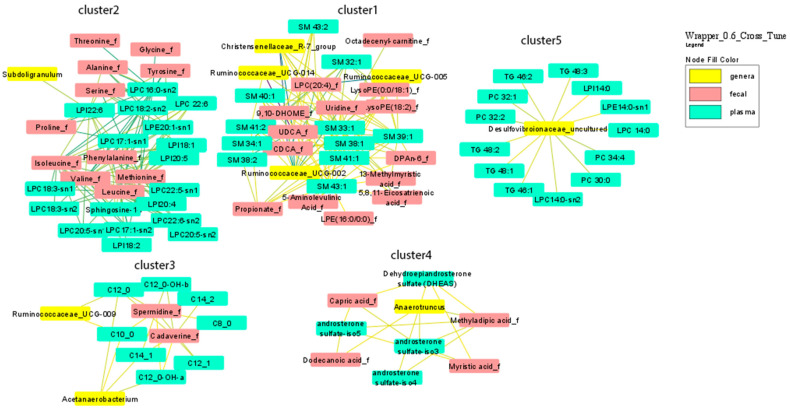
Network analysis of correlation for the multi-omics clusters.

Figure 1 shows the network deriving from the correlation network analysis with a cut-off point value of 0.6. Ninety-four nodes and 263 edges characterized the resulting network, which displays the most relevant correlations deriving from the SGCCA between the different multi-omics data. The network shows five distinct multi-omics clusters, including a total number of 9 genera, 31 fecal metabolites and 41 plasma metabolites. A detailed description of the network and cluster characteristics is given in Supplementary Table S3 and Figure S1.

In the tri-omics cluster one, we observed a hub of connected components, mainly constituted by a fecal primary bile acid [chenodeoxycholic acid (CDCA)] and its derivative secondary bile acid, ursodeoxycholic acid (UDCA), fecal propionate, fecal derivatives of arachidonic acid, as well as, fecal LPC 16:0 and LPC 20:4, that were negatively correlated with three uncultured genera from the Ruminococcaceae family and Christensenellaceae R7 group. These microbial genera were also positively correlated with several plasma sphingomyelins (SMs) with one and two double bonds. Cluster two mainly consisted of fecal amino acids positively correlated with plasma species of LPCs (16:0, 17:1, 18:2, 18:1, 18:3, 20:5, 22:6) and LPIs (18:1, 18:2, 20:4). Plasma levels of LPI 22:6 were also negatively correlated with Subdoligranum. The multi-omics cluster three was constituted by plasma levels of medium/long-chain mainly saturated acylcarnitines (C8:0, C10:0, C12:0, C12:1, C14:1, C14:2) found to be positively correlated with fecal levels of spermidine and cadaverine. Plasma levels of C10:0, C12:0, C14:1, C12:0-OH were also negatively correlated with two genera from the Ruminococcaceae family (Ruminococcaceae UCG09 group and *Acetanaereobacterium*). The multi-omics cluster four was formed by fecal fatty acids such as myristic acid, methyladipic acid, capric acid and dodecanoic acid that were positively correlated with plasma levels of different androsterones, including dehydroepiandrosterone sulfate (DHEAS), and with a member of Firmicutes (*Anaerotruncus*). The multi-omics cluster five was a dual-omics hub of plasma levels of phosphocholines (PCs 32:2, 32:1, 34:4, 30:0), triglycerides (TGs 46:1, 46:2, 48:1, 48:2, 48:3), lysophosphatidylcholine (LPC) 14:0, lysophosphatidylethanolamine (LPE) 14:0 and lysophosphatidylinositol (LPI) 14:0 negatively correlated with a genus from the Desulfovibrionaceae family. Better visualization of the direction of each correlation is shown in Supplementary Figures S2–S4 [12].

### 3.2. Associations between Multi-Omics Scores and Cardiometabolic Risk Factors

The linear regression analysis of associations between the five multi-omics scores (one for each cluster) and cardiometabolic parameters are shown in Table 2.

Significant positive associations of the multi-omics cluster one score with total and LDL cholesterol levels were observed. Significant positive associations were also found between the multi-omics cluster five score and levels of cholesterol (total, LDL and VLDL) and triglycerides.

**Figure 2:**
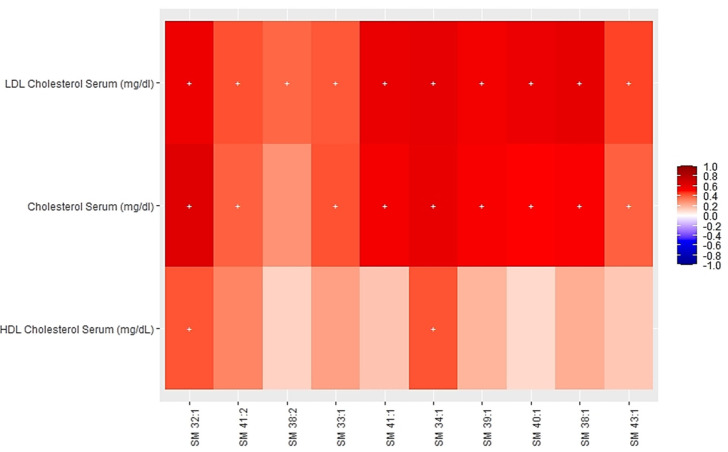
Heatmap of Pearson partial correlations between individual components of the multi-omics cluster 1 and cardiometabolic risk factors.

**Figure 3:**
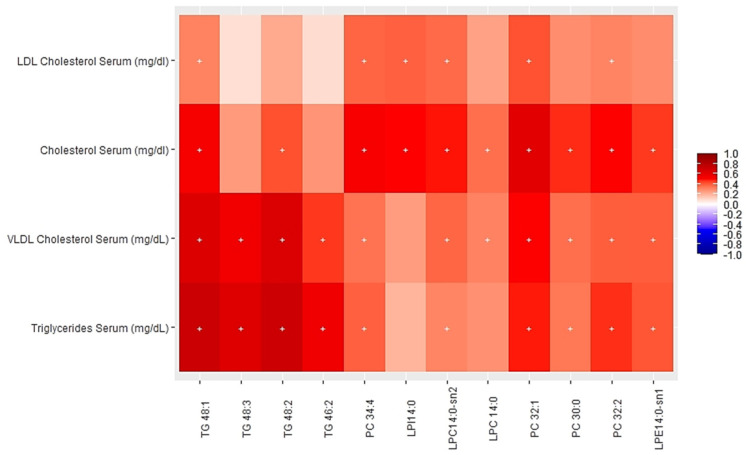
Heatmap of Pearson partial correlations between individual components of the multi-omics cluster 5 and selected cardiometabolic risk factor.

Figure 2 and Figure 3 show the heatmaps of Pearson correlations between individual components of the multi-omics clusters one and five, and cardiometabolic parameters.

Figure 2 shows significant positive correlations between specific plasma levels of certain SMs (32:1, 34:1, 38:1, 39:1, 40.1, 41:1, 43:1) from the multi-omics cluster one and cholesterol (total and LDL) levels as well as a positive correlation of SM32:1 and SM34:1 with HDLc. Figure 3 shows significant positive correlations of PCs (30:0, 32:1, 32:2, 34:4), LPC 14:0, LPI 14:0, LPE 14:0 and different TGs species (46:2, 48:1, 48:2, 48:3) from cluster five with serum levels of cholesterol (total, LDL, VLDL) and triglycerides.

## 4. Discussion

In this secondary analysis conducted in the METADIET study, we identified a network of correlations between microbial genera and specific fecal and circulating metabolites that constituted five different multi-omics clusters. Two of the identified clusters, and especially plasma lipid species, were associated with cardiometabolic risk factors highlighting their importance on cardiometabolic health [17]. Despite the absence of a direct correlation between the identified microbial genera and cardiometabolic health features, their co-occurrence with their microbial functional readout represented by fecal metabolites and known secondary biomarkers of cardiometabolic health in plasma offers new insights for further mechanistic studies.

Cluster one demonstrated a cross-talk between circulating SM species and fecal bile acids and arachidonic acid derivatives in which members from the Ruminococcaceae and Christensenellaceae families were involved. The correlation of these interrelated metabolic components with total and LDL cholesterol adds evidence on the role of SMs on cholesterol metabolism. Indeed, SMs are the most abundant sphingolipids in lipoproteins, such as LDL, and their levels in plasma have been previously associated with cardiovascular disease risk [18]. SMs also play a role in cholesterol homeostasis [19] by affecting LDL’s ability to bind surface receptors and subsequent internalization [19]. A growing body of evidence regarding their favorable interaction with sterols indicates SMs as a potential key regulator of cholesterol distribution within cellular membranes. Interestingly, the *Christensenellaceae R7* group and *Ruminococcaceae UCG002*, which we found to be positively correlated with these SM species, have both been negatively associated with VLDL diameter in an observational study conducted in The Netherlands [20]. Similarly, other studies have also found negative associations of the Christensenellaceae family with total and LDL cholesterol and apolipoprotein particles [21,22], suggesting its role in overall cholesterol homeostasis. Whether these associations could be partially explained by SMs is a hypothesis that needs further investigation. At the same time, we observed negative correlations between the *Christensenellaceae R7* group and *Ruminococcaceae UCG002* with fecal bile acids CDCA and its microbial derivative UDCA [23], supporting the mediation of the microbial activity on fecal bile acids in cluster two. Reinforcing our findings of the potential involvement of this cluster in cholesterol metabolism, a recent study conducted in an Italian elderly cohort was able to characterize a healthier metabolic profile by Christensenellaceae-enterotype, which was also associated with an improved visceral lipid composition, as well as with a trend towards lower serum levels of CDCA [24], whose harmful impact on cholesterol metabolism has been previously demonstrated [25]. On the contrary, the therapeutical benefits of secondary bile acid UDCA in reducing cholesterol solubilization in blood has been demonstrated [23]. In this cluster, circulating levels of SMs were also positively correlated with fecal 13-methylmyristic acid, also known as 13-methytetradecanoic acid (13-MTD). 13-MTD is a saturated iso-fatty acid that derives from microbial fermentation and has been extensively studied for its apoptotic properties, especially in certain cancer cells [26], other than being proposed as a marker of adipose tissue turnover [27]. In this same cluster, fecal levels of the SCFA propionate were also related to plasma levels of different SM species (38:2, 41:2) and Ruminococcaceae UCG-002. Despite the fact that no specific genera from Ruminococcaceae have been previously addressed to propionate production pathways [28], we could speculate its indirect involvement in propionate metabolism.

In cluster two, fecal amino acids, including microbial-derived BCAAs, aromatic amino acids and proline, were correlated with plasma LPC and LPI species, and LPI 22:6 was negatively correlated with *Subdoligranulum*. The genus *Subdoligranulum*, from the Ruminococcaceae family, is a Gram-negative, strictly anaerobe and butyrate-producer [29], which has been recently proposed as beneficial bacteria for metabolic health [30]. Strong evidence supports a relationship between circulating BCAAs and cardiometabolic diseases [31,32], and a similar pattern seems to be reflected in feces regarding the association of fecal BCAAs with insulin resistance [33]. However, in our study, fecal BCAAs were not correlated with cardiometabolic risk parameters. The positive correlation we found between fecal levels of BCAAs and LPCs and LPI species in plasma is not supported by previous findings; therefore, the potential intermediation of this metabolic interaction in cardiometabolic health needs further mechanistic studies. 

In cluster three, plasma levels of long-chain acylcarnitines were negatively correlated with fecal levels of the polyamines cadaverine and spermidine, as well as negatively correlated with members of the Ruminococcaceae family (*Acetanaerobacterium* and *Ruminococcaceae UCG-009*). Plasma levels of long (C12, C14) and medium (C8, C10) chain acylcarnitines are associated with β oxidation of fatty acids [34]. A similar pattern of correlations between medium and long-chain acylcarnitines and both spermidine and cadaverine were associated with increased BMI in the Northern Finland Birth Cohort, while some acylcarnitines were correlated with an unknown genus from the Firmicutes phylum [35]. *Acetanaerobacterium* and *Ruminococcaceae UCG-009* are both butyrate-producers belonging to the Ruminococcaceae family and are known for their potential protective role in the metabolism of high-fat diet-induced obese mice [36]. Different members of the Ruminococcaceae family have been previously correlated with lower levels of medium-chain acylcarnitines in a randomized crossover clinical trial comparing vegetarians versus meat-eaters [37]. Other studies have also demonstrated the ability of some bacteria to ferment cadaverine for butyrate production [38], partially explaining our observations about the co-appearance of the Ruminococcaceae members with fecal cadaverine. Therefore, there might be a potential involvement of this multi-omics profile of butyrate-producers in energy homeostasis through the regulation of fatty acids oxidation. 

In cluster four, *Anaerotruncus* from the family of Clostridiaceae was correlated with fecal levels of dodecanoic acid and methyladipic acid, as well as with plasma levels of different steroid species of androsterone sulfate, including DHEAS. Previous studies have observed the involvement of *Anaerotruncus* in the biosynthesis of both steroids and terpenoids in colorectal cancer [39]. Although a recent in vivo study found a relationship between lower plasma concentrations of DHEAS and impaired glucose tolerance [40], we did not find any significant correlation between plasma levels of steroids and cardiometabolic parameters.

Finally, a dual-omics profile was identified in our study. To our knowledge, this is the first study finding negative correlations of plasma phospholipid species and TGs with 46 or 48 carbon atoms and <3 double bonds with bacteria from the Desulfovibrioaceae family. Previous evidence suggests that the Desulfovibrionaceae family members might have an adverse effect on dyslipidemia, even if the biological mechanisms behind this association remain still unknown [41]. Therefore, our results in relation to the correlations of lipid species with serum cholesterol (total, LDL, VLDL) and triglycerides support the involvement of this microbial-host cross-talk in lipid metabolism and transport.

The present study has some strengths that deserve to be mentioned, like being one of the few studies analyzing the microbial-host cross-talk by using the combination of three different omics, the use of a multi-platform metabolomics analysis, which allowed to combine targeted and untargeted analytical approaches, in order to cover a wide range of metabolites. Furthermore, the availability of a pretty uniform population characterized by MetS allowed us to relate the microbial-host cross-talk with cardiometabolic risk factors. Regarding limitations, this is a cross-sectional study in a small-sized population, and no causal relationship between the microbiome and the metabolome or between the metabolome and the panel of cardiometabolic risk factors can be inferred. Due to the fact that the implemented model is right below the limits to be considered as a robust model, some of our findings could be the result of spurious correlations. Furthermore, the use of 16S rRNA sequencing does not allow unraveling of the taxonomical composition of gut microbiota up to the strain level. A further limitation of the study is the lack of a “healthy” control group. This may have partially accounted for the lack of significant associations between the multi-omics scores and cardiometabolic measures such as insulin and HOMA-IR due to their low variation among the study population. As well, we evaluated a sample of individuals with MetS that could limit the generalizability of our results to other populations. Other larger studies would be necessary to confirm our novel findings, ideally, prospectively.

## 5. Conclusions

In conclusion, this study demonstrated a network of five host-microbial cross-talk profiles in adults with MetS. A total of 41 plasma and 31 fecal metabolites, as well as 9 microbial genera, were identified in 5 clusters. Plasma levels of different lipid species (SMs, LPCs, LPIs and long-chain acylcarnitines) were mainly correlated with members from the Ruminococcaceae and Christenseneellaceae families, as well as with fecal bile acids, BCAAs, AAs and polyamines. The plasma lipid correlations with serum total LDL and VLDL cholesterol and triglycerides levels suggest a link between the microbial-host cross-talk and cardiometabolic health.

## Figures and Tables

**Table 1:** General characteristics of study participants.

Characteristics	Baseline *n* = 47
Age (years)	50.6 (48.6, 52.6)
Women *n* (%)	30 (63.8%)
BMI (Kg/m^2^)	30.5 (29.9, 31.2)
Waist Circumference (cm)	102.1 (99.3, 104.8)
SBP (mmHg)	135.1 (131.7, 138.6)
DBP (mmHg)	85.0 (82.3, 87.7)
Total Cholesterol (mg/dL)	215.3 (206.1, 224.5)
LDLc (mg/dL)	135.8 (128.1, 143.6)
HDLc (mg/dL)	50.5 (47.6, 53.5)
VLDLc (mg/dL)	28.1 (24.7, 31.5)
Triglycerides (mg/dL)	147.7 (126.3, 169.2)
Glucose (mg/dL)	100.2 (96.5, 103.9)
Insulin (mcUI/mL)	13.4 (11.3, 15.5)
HOMA-IR	3.3 (2.8, 3.9)

All values are given as means (95% CI). Abbreviations: BMI; body mass index, SBP; systolic blood pressure, DBP; diastolic blood pressure, LDLc; low-density lipoprotein cholesterol, HDLc; high-density lipoprotein cholesterol, VLDLc; very-low-density lipoprotein cholesterol, HOMA-IR; homeostatic model assessment of insulin resistance.

**Table 2:** Linear regression analysis examining the associations of 1-SD increase of multi-omics scores with cardiometabolic risk factors.

	Multi-Omic Score 1		Multi-Omic Score 2		Multi-Omic Score 3		Multi-Omic Score 4		Multi-Omic Score 5	
Factor	Mean ± SE	*p* ^1^	Mean ± SE	*p* ^1^	Mean ± SE	*p* ^1^	Mean ± SE	*p* ^1^	Mean ± SE	*p* ^1^
Cholesterol (mg/dL)	1.666 ± 0.435	0.001	0.249 ± 0.087	0.070	0.099 ± 0.215	0.983	0.121 ± 0.311	0.743	0.621 ± 0.148	0.0003
LDLc (mg/dL)	1.269 ± 0.336	0.005	0.161 ± 0.0692	0.083	0.062 ± 0.166	0.983	0.079 ± 0.239	0.743	0.327 ± 0.126	0.032
HDLc (mg/dL)	0.374 ± 0.149	0.053	0.0452 ± 0.029	0.330	−0.001 ± 0.068	0.983	0.089 ± 0.098	0.743	0.0326 ± 0.055	0.803
VLDLc (mg/dL)	−0.068 ± 0.179	0.755	0.036 ± 0.033	0.495	0.030 ± 0.076	0.983	0.050 ± 0.110	0.743	0.224 ± 0.052	0.0003
Triglycerides (mg/dL)	0.373 ± 1.186	0.755	0.234 ± 0.221	0.495	0.222 ± 0.505	0.983	−0.498 ± 0.728	0.743	1.420 ± 0.351	0.0003
Glucose (mg/dL)	0.358 ± 0.191	0.170	0.087 ± 0.035	0.083	0.132 ± 0.0824	0.983	0.116 ± 0.121	0.743	0.043 ± 0.069	0.803
Insulin (mcUI/mL)	0.088 ± 0.114	0.738	0.007 ± 0.021	0.838	−0.028 ± 0.0486	0.983	−0.062 ± 0.070	0.743	−0.010 ± 0.040	0.803
HOMA IR	0.034 ± 0.029	0.472	0.004 ± 0.005	0.656	−0.0005 ± 0.012	0.983	−0.008 ± 0.018	0.743	−0.003 ± 0.010	0.803
SBP (mmHg)	0.077 ± 0.191	0.755	0.019 ± 0.036	0.749	0.105 ± 0.080	0.983	−0.117 ± 0.117	0.743	0.019 ± 0.067	0.803
DBP (mmHg)	0.065 ± 0.148	0.755	0.002 ± 0.028	0.951	0.006 ± 0.063	0.983	0.045 ± 0.091	0.743	0.028 ± 0.052	0.803

Values are given as means ± standard error. Model was adjusted for sex, age and BMI. Abbreviations, DBP; diastolic blood pressure, LDLc; low-density lipoprotein cholesterol, HDLc; high-density lipoprotein cholesterol, VLDLc; very-low-density lipoprotein cholesterol, HOMA-IR; homeostatic model assessment of insulin resistance, SBP; systolic blood pressure, DBP; diastolic blood pressure. ^1^ Adjusted with the Benjamini-Hochberg False Discovery Rate method.

## Data Availability

Further data will be provided under request to the authors.

## Supplementary Materials

The following are available online at https://www.mdpi.com/article/10.3390/nu13124318/s1, Figure S1. Description of the connected components of the network; Figure S2. Heatmap of correlations from the network similarity matrix between selected plasma and fecal metabolites; Figure S3. Heatmap of correlations from the network similarity matrix between selected plasma metabolites and microbial genera; Figure S4. Heatmap of correlations from the network similarity matrix between selected fecal metabolites and microbial genera; Supplementary Table S1. List of fecal metabolites analyzed; Supplementary Table S2. List of plasma metabolites analyzed; Supplementary Table S3. Summary statistics of the network analysis.
