# Supplementary material for: Examining the Interaction of the Gut Microbiome with Host Metabolism and Cardiometabolic Health in Metabolic Syndrome
Source: Nutrients. 2021 Nov 29;13(12):4318. doi: 10.3390/nu13124318 (PMC8706982; doi:10.3390/nu13124318)
Supplement: Supplementary file 1 [file nutrients-13-04318-s001.zip › nutrients-1455991-supplementary.pdf]

Supplementary Material

Supplemental Methods

Fecal Metabolomics

An initial amount of 10-15 mg of lyophilized feces was used for the sample preparation Also, there was a previous step of metabolites extraction including a sonication and homogenization with either 500 µL of PBS in case of NMR or 20 µL of an internal standard in case of the other chromatographic platforms. After the extraction of the water components from the fecal matter, 200µl of fecal aqueous phase were diluted in 400µl of PBS in D2O (pH=7.4, 0.05M, TSP 1.48mM for diluted concentration of 1mM) and placed into a 5mm o.d. NMR tube. ^{1}H-NMR spectra were recorded at 300K on an Advance III 600 spectrometer (Bruker, Germany) operating at a proton frequency of 600.20MHz using a 5mm PBBO broadband gradient probe. The acquired NMR was compared to references of pure compounds from the metabolic profiling AMIX spectra database (Bruker®), HMDB, and Chenomx databases for metabolite identification. We assigned metabolites by 1H–1H homonuclear correlation (COSY and TOCSY) and 1H–13C heteronuclear (HSQC) 2D-NMR experiments and by correlation with pure compounds run in-house. After pre-processing, specific 1H-NMR regions identified in the spectra were integrated using the AMIX 3.9 software package.

LC-qTOF was used to determine bile acids, amino acids and its derivatives. The chromatographic separation of bile acids was performed on a Kinetex EVO C18 (150x2.1mm) column and bile acid species were assigned by direct comparison with commercial standards. At the same time, the quantification of amino acids was achieved by chromatographic separation of performed on a ACQUITY UPLC HSS T3 Column, and the metabolites were identified by direct comparison with commercial standards. The chromatographic behavior and presence of possible interferences were based on a methodology previously described[1].

The untargeted approach was focused on the detection of phenolic compounds, fatty acids and acylcarnitines. After a previous homogenization step with an internal standard reference for the class of the sought metabolites, the chromatographic separation was achieved with a gradient elution using milli-Q water (0.05% formic acid) and methanol (0.05% formic acid) on a reverse phase AQUITY BEH C18 (100x2.1mm) column from Waters®. The qTOF mass spectrometer operates in scan mode both in positive and negative electrospray ionization on two separate chromatographic runs. In addition, a target tandem mass spectrum (MS/MS) was acquired for QC sample at 20 eV for deconvoluted features. MassHunter qualitative analyses were used for metabolite profiling using "find by formula" algorithm from an in-house database created from the human fecal metabolome database from HMDB. The resulting tentative matched entities were refined manually by comparing the experimental MS/MS spectra, retention time with the information on database or pure standards when available, also published in relation to the main phenolic metabolites, fatty acids and other fecal metabolites. After metabolite screening, a total of 132 compounds were found and used in MassHunter quantitative analysis for chromatographic peak extraction and integration. The area of each metabolite was normalized by the area of the corresponding internal standard compound based on their structural similarity and retention time proximity. Finally, the matrix containing the semi-quantitative information for these metabolites was normalized by the exact sample weight.

Plasma Metabolomics

In case of TMAO determinations, aliquots of 50 µl were mixed with 10 µl of internal standard, 75 µl of 50mM tert-Butyl bromoacetate in ACN and 10 µl of 70% ammonium hydroxide. Samples were vortexed for 1 min and derivatized for 30 minutes at room temperature. A volume of 50 µl of 1% Formic acid in ACN was added. Samples were vortexed and centrifuged for 5 minutes at 15000 rpm and 4ºC. Supernatants were

transferred to glass vials for their analysis. Mobile phase A was 10% acetonitrile/90% water and B was 90% acetonitrile/10% water, and both with 10 mM ammonium formate and 0.125% formic acid. The column temperature was set at room temperature and the injection volume was 1 µl.

In case of acylcarnitines quantification, 30 µl of plasma were mixed with 270 µL of 100% methanol containing the set of labelled internal standards. The mixture was vortexed for 15 s and centrifuged during 10 min at 4700 rpm at 4ºC. The supernatant was transferred into a new plate and injected on LC-MS/MS. The extraction was carried out with a semi-automated process using Agilent Bravo Automated Liquid Handling Platform. The chromatographic separation was performed with an isocratic gradient of mobile phase B was 100% methanol with 0.1% formic acid over 13 minutes. The column temperature was set at 20 ºC and the injection volume was 1 µL.

For the extraction of more hydrophobic lipids, a liquid-liquid extraction with chloroform:methanol (2:1) based on Folch procedure was performed by adding four volumes of chloroform:methanol (2:1) containing internal standard mixture (Lipidomic SPLASH) to serum. Then, the samples were mixed and incubated at -20ºC for 30 minutes. Afterward, water with NaCl (0.8 %) was added and mixture was centrifuged at 15,000 rpm. The lower phase was recovered, evaporated to dryness and reconstituted with methanol:methyl-tert-butyl ether (9:1) and analyzed by UHPLC-qTOF (model 6550 of Agilent, USA) in positive electrospray ionization mode. The chromatographic method consists of elution with a ternary mobile phase containing water, methanol and 2-propanol with 10mM ammonium formate and 0.1% formic acid. The stationary phase was a C18 column (Kinetex EVO C18 Column, 2.6 µm, 2.1 mm X 100 mm) that allows the sequential elution of the more hydrophobic lipids such as lysophospholipids,

sphingomyelins, phospholipids, diglycerides, triglycerides and cholesteryl esters, among others.

The identification of lipid species was performed by matching their accurate mass and tandem mass spectrum, when available, to Metlin-PCDL from Agilent containing more than 40,000 metabolites and lipids. In addition, chromatographic behavior of pure standards for each family and bibliographic information was used to ensure their putative identification.

Sample preparation was based on[1] to obtain volatile fatty acids methyl ester derivatives (FAMEs). Briefly, 50 µl of plasma samples were mixed with IS solution, chloroform and methanolic HCl and incubated at 80 ºC for 2 hours. Afterward, obtained FAMEs were extracted by a liquid-liquid extraction using hexane before being injected intotheGC-MS system.

Chromatographic analysis was based on[2] to determine the 37 FAMEs included in Food Industry FAME Mix. Briefly, FAMEs were separated on HP-88 (100 m x 250 µm x 0.25 µm) column using a temperature program between 140 and 240 ºC at 1mL/min using He as carrier gas. Ionization was carried out by electronic impart (70 eV) and the mass analyzer operates on Selected Ion Monitoring mode (SIM).

Serotonin was analyzed starting from 50 µL of plasma, which was aliquoted to a 1.5 ml Eppendorf tube and mixed with 5 µL of internal standard (Serotonin-d4 at 1 µg/mL) and 245 µL of acetonitrile. Samples were vortexed and centrifuged for 5 minutes at 15000 rpm and 4ºC. Supernatants were transferred to glass vials for analysis. The analytical column used for serotonin was Luna Omega 1.6 µm Polar C18 (100 x 2.1 mm) (Phenomenex, Torrance, CA).

The chromatographic separation was performed with a linear gradient to 99% mobile phase B of 0.1% formic acid in acetonitrile. The column temperature was set at 35 ºC and the injection volume was 5 µL.

Amino acids determination started from 8 µL of plasma mixed with 32 µL of internal standard (Metabolomics amino acid mix labeled standard from Cambridge Isotopes) in MeOH. Samples were vortexed and centrifuged for 5 minutes at 15000 rpm and 4ºC. Supernatants (30 µL) were transferred to a new tube and evaporated in a SpeedVac at 45 ºC. The analytical column used was ACQUITY UPLC HSS T3 Column, 1.7 µm, 2.1 mm x 150 mm (Waters, Milford, MA, USA). Samples were reconstituted with 30 µL of borate buffer and were derivatized using AccQ-Tag reagent from Waters following manufacturing protocol. The chromatographic separation was performed with a linear gradient to 70% mobile phase B (0.1% formic acid in acetonitrile) over 13 minutes and an isocratic gradient to 87% of mobile phase A (0.1% formic acid in water) over 2 minutes. The column temperature was set at 40 ºC and the injection volume was 2 µL. The MRM transitions for amino acids and metabolites in all cases the precursor ion was the [derivatized amino acid + H]+ and the main daughter ion was 171 m/z. The chromatographic behavior and presence of possible interferences was based on the methodology described by Wang et al[1].

Supplementary Table S1: List of fecal metabolites analyzed

Name	Platform	Approach
1,3-Dihydroxyacetone	NMR	Targeted
2-hydroxybutyrate	NMR	Targeted
2-Oxocaproate	NMR	Targeted
Acetate	NMR	Targeted
Arabinose	NMR	Targeted
Butyrate	NMR	Targeted
Caproate	NMR	Targeted
Cholate	NMR	Targeted
Choline	NMR	Targeted
Dimethylamine	NMR	Targeted
Ethanol	NMR	Targeted
Ethanolamine	NMR	Targeted
Formate	NMR	Targeted
Fumarate	NMR	Targeted
Galactonate	NMR	Targeted
Glucose	NMR	Targeted
Glycerol	NMR	Targeted
Hydroxyacetone	NMR	Targeted
Hypoxanthine	NMR	Targeted
Isobutyrate	NMR	Targeted
Malate	NMR	Targeted
Malonate	NMR	Targeted
Methanol	NMR	Targeted
Methionine Sulphoxide	NMR	Targeted
Methylamine	NMR	Targeted
Methylguanidine	NMR	Targeted
Propionate	NMR	Targeted
Ribose	NMR	Targeted
Succinate	NMR	Targeted
Trimethylamine	NMR	Targeted
UDP-Glucoronate	NMR	Targeted
Uracil	NMR	Targeted
Uridine	NMR	Targeted
Urocanate	NMR	Targeted
Valerate	NMR	Targeted
Xanthine	NMR	Targeted
Xylose	NMR	Targeted
5-Aminolevulinic Acid	LC-qTOF	Targeted
ABA	LC-qTOF	Targeted
AIBA	LC-qTOF	Targeted
Alanine	LC-qTOF	Targeted
Arginine	LC-qTOF	Targeted
Asparagine	LC-qTOF	Targeted
Aspartic acid	LC-qTOF	Targeted
BAIBA	LC-qTOF	Targeted
bAlanine	LC-qTOF	Targeted
CA	LC-qTOF	Targeted
Cadaverine	LC-qTOF	Targeted
Carnosine	LC-qTOF	Targeted
CDCA	LC-qTOF	Targeted
Citrulline	LC-qTOF	Targeted
DCA	LC-qTOF	Targeted
Ethanolamine	LC-qTOF	Targeted
GABA	LC-qTOF	Targeted
GCA	LC-qTOF	Targeted

GCDCA	LC-qTOF	Targeted
GDCA	LC-qTOF	Targeted
GLCA	LC-qTOF	Targeted
Glutamic acid	LC-qTOF	Targeted
Glutamine	LC-qTOF	Targeted
Glycine	LC-qTOF	Targeted
GUDCA	LC-qTOF	Targeted
HDCA	LC-qTOF	Targeted
Hipotaurine	LC-qTOF	Targeted
Histidine	LC-qTOF	Targeted
Homocitrulline	LC-qTOF	Targeted
Hydroxyproline trans	LC-qTOF	Targeted
Isoleucine	LC-qTOF	Targeted
Kynurenine	LC-qTOF	Targeted
L-2-Aminoadipic Acid	LC-qTOF	Targeted
LCA	LC-qTOF	Targeted
Leucine	LC-qTOF	Targeted
Lysine	LC-qTOF	Targeted
Methionine	LC-qTOF	Targeted
Ornithine	LC-qTOF	Targeted
Phenylalanine	LC-qTOF	Targeted
Pipecolic Acid	LC-qTOF	Targeted
Proline	LC-qTOF	Targeted
Putrescine	LC-qTOF	Targeted
Sarcosine	LC-qTOF	Targeted
Serine	LC-qTOF	Targeted
Serotonin	LC-qTOF	Targeted
Spermidine	LC-qTOF	Targeted
Taurine	LC-qTOF	Targeted
TCA	LC-qTOF	Targeted
TCDCA	LC-qTOF	Targeted
TDCA	LC-qTOF	Targeted
Threonine	LC-qTOF	Targeted
TLCA	LC-qTOF	Targeted
Tryptophan	LC-qTOF	Targeted
TUDCA	LC-qTOF	Targeted
Tyrosine	LC-qTOF	Targeted
UDCA	LC-qTOF	Targeted
Valine	LC-qTOF	Targeted
(S)-14-Methylhexadecanoic acid	LC-qTOF	Untargeted
10Z-Heptadecenoic acid	LC-qTOF	Untargeted
10Z-Nonadecenoic acid	LC-qTOF	Untargeted
11Z-Eicosenoic acid	LC-qTOF	Untargeted
12,13-DHOME	LC-qTOF	Untargeted
13-HODE	LC-qTOF	Untargeted
13-Methylmyristic acid	LC-qTOF	Untargeted
1-Stearoylglycerophosphoinositol	LC-qTOF	Untargeted
2-Hydroxyhexadecanoic acid	LC-qTOF	Untargeted
2-Hydroxymyristic acid	LC-qTOF	Untargeted
2-Hydroxyphenylacetic acid	LC-qTOF	Untargeted
2-Hydroxystearic acid	LC-qTOF	Untargeted
2-Methylglutaric acid	LC-qTOF	Untargeted
2-Oleoylglycerophosphocholine	LC-qTOF	Untargeted
3-(3′-Hydroxyphenyl)propionic acid	LC-qTOF	Untargeted
3-(3′-Hydroxyphenyl)propionic acid-4′-O-glucuronide	LC-qTOF	Untargeted

3-(3′-Methoxy-4′-hydroxyphenyl)propionic acid	LC-qTOF	Untargeted
3-(4′-Hydroxyphenyl)propionic acid-3′-O-glucuronide	LC-qTOF	Untargeted
3-(Phenyl)propionic acid-3′-sulfate	LC-qTOF	Untargeted
3,4-Dihydroxyphenylacetic acid	LC-qTOF	Untargeted
3′-Methoxy-4′-hydroxyphenylacetic acid	LC-qTOF	Untargeted
3-Hydroxy-3-(3-hydroxyphenyl)propionic acid	LC-qTOF	Untargeted
3-Hydroxybenzoic acid (3-OH-BA)	LC-qTOF	Untargeted
3-Phenylpropionic acid	LC-qTOF	Untargeted
4-Hydroxybenzoic acid (4-OH-BA)	LC-qTOF	Untargeted
5-(3′,4′-Dihydroxyphenyl)-γ-valerolactone	LC-qTOF	Untargeted
5-(3′-Hydroxyphenyl)-γ-valerolactone	LC-qTOF	Untargeted
5,8,11-Eicosatrienoic acid	LC-qTOF	Untargeted
5-Hydroxyhexanoic acid	LC-qTOF	Untargeted
7Z,10Z-Hexadecadienoic acid	LC-qTOF	Untargeted
8,11,14-Eicosatrienoic acid	LC-qTOF	Untargeted
9,10-DHOME	LC-qTOF	Untargeted
9-HODE	LC-qTOF	Untargeted
9Z-Eicosenoic acid	LC-qTOF	Untargeted
Acetaminophen	LC-qTOF	Untargeted
Adrenic acid	LC-qTOF	Untargeted
Alpha-Linolenic acid	LC-qTOF	Untargeted
Arachidic acid	LC-qTOF	Untargeted
Arachidonic acid	LC-qTOF	Untargeted
Arginyl-Isoleucine	LC-qTOF	Untargeted
Arginyl-Phenylalanine	LC-qTOF	Untargeted
Azelaic acid	LC-qTOF	Untargeted
Behenic acid	LC-qTOF	Untargeted
Benzoic acid (BA)	LC-qTOF	Untargeted
Bilirubin	LC-qTOF	Untargeted
Caffeic acid 3-O-β-d-glucuronide (CA-3-Glu)	LC-qTOF	Untargeted
Caffeic acid 4-O-β-d-glucuronide (CA-4-Glu)	LC-qTOF	Untargeted
Caffeine	LC-qTOF	Untargeted
Capric acid	LC-qTOF	Untargeted
Chlorogenic acid	LC-qTOF	Untargeted
Chlorogenic acid-iso	LC-qTOF	Untargeted
Decanoyl-carnitine/Fumaryl-carnitine	LC-qTOF	Untargeted
Dehydroepiandrosterone sulfate	LC-qTOF	Untargeted
Dihomolinoleic acid	LC-qTOF	Untargeted
Dihydrocaffeicacid 3-O-β-d-glucuronide (DHCA-Glu)	LC-qTOF	Untargeted
Docosahexaenoic acid	LC-qTOF	Untargeted
Docosapentaenoic acid (22n-3)	LC-qTOF	Untargeted
Docosapentaenoic acid (22n-6)	LC-qTOF	Untargeted
Docosatrienoic acid	LC-qTOF	Untargeted
Dodecanoic acid	LC-qTOF	Untargeted
Dodecanoyl-carnitine	LC-qTOF	Untargeted

D-Urobilinogen+Urobilin	LC-qTOF	Untargeted
Eicosadienoic acid	LC-qTOF	Untargeted
Eicosapentaenoic acid	LC-qTOF	Untargeted
Elaidic acid	LC-qTOF	Untargeted
Enterodiol	LC-qTOF	Untargeted
Enterolactone	LC-qTOF	Untargeted
Ferulic acid/Isoferulic acid (IsoFA)	LC-qTOF	Untargeted
Free carnitine	LC-qTOF	Untargeted
gamma-Glutamyltryptophan	LC-qTOF	Untargeted
Gamma-Linolenic acid	LC-qTOF	Untargeted
Gluconic acid	LC-qTOF	Untargeted
Glutaminylglutamine	LC-qTOF	Untargeted
Heptadecanoic acid	LC-qTOF	Untargeted
Hexadecanoyl-carnitine	LC-qTOF	Untargeted
Hexadecenyl-carnitine	LC-qTOF	Untargeted
Hippuric acid (HA)	LC-qTOF	Untargeted
Histidinyl-Isoleucine	LC-qTOF	Untargeted
Histidinyl-Phenylalanine	LC-qTOF	Untargeted
Histidinyl-Proline	LC-qTOF	Untargeted
Linoelaidic acid	LC-qTOF	Untargeted
Linoleic acid	LC-qTOF	Untargeted
LysoPC(20:4(8Z,11Z,14Z,17Z))	LC-qTOF	Untargeted
LysoPE(0:0/18:1(11Z))	LC-qTOF	Untargeted
LysoPE(15:0/0:0)	LC-qTOF	Untargeted
LysoPE(16:0/0:0)	LC-qTOF	Untargeted
LysoPE(18:0/0:0)	LC-qTOF	Untargeted
LysoPE(18:2(9Z,12Z)/0:0)	LC-qTOF	Untargeted
Mesobilirubinogen	LC-qTOF	Untargeted
Methyl 3-phenylpropanoate	LC-qTOF	Untargeted
Methyladipic acid	LC-qTOF	Untargeted
Mevalonic acid	LC-qTOF	Untargeted
Myristic acid	LC-qTOF	Untargeted
N-Acetylleucine	LC-qTOF	Untargeted
N-Acetyl-L-methionine	LC-qTOF	Untargeted
N-Acetyl-L-phenylalanine	LC-qTOF	Untargeted
N-acetyltryptophan	LC-qTOF	Untargeted
Nervonic acid	LC-qTOF	Untargeted
Nonadecanoic acid	LC-qTOF	Untargeted
Octadecadienyl-carnitine	LC-qTOF	Untargeted
Octadecenyl-carnitine	LC-qTOF	Untargeted
Octadecenyl-carnitine	LC-qTOF	Untargeted
Octenoyl-carnitine	LC-qTOF	Untargeted
Oleanolic acid	LC-qTOF	Untargeted
Oleic acid	LC-qTOF	Untargeted
Palmitelaidic acid	LC-qTOF	Untargeted
Palmitic acid	LC-qTOF	Untargeted
Palmitoleic acid	LC-qTOF	Untargeted
Pantothenic acid	LC-qTOF	Untargeted
p-Cresol	LC-qTOF	Untargeted
p-Cresol sulfate	LC-qTOF	Untargeted
Pentadecanoic acid	LC-qTOF	Untargeted
Pentadecanoyl-carnitine/isopentadecanoyl-carnitine	LC-qTOF	Untargeted
Phenylacetic acid (PA)	LC-qTOF	Untargeted
Phytanic acid	LC-qTOF	Untargeted
Stearic acid	LC-qTOF	Untargeted

Stercobilin	LC-qTOF	Untargeted
Suberic acid	LC-qTOF	Untargeted
Tetracosanoic acid	LC-qTOF	Untargeted
Tetradecanoyl-carnitine	LC-qTOF	Untargeted
Threoninyl-Phenylalanine	LC-qTOF	Untargeted
Tricosanoic acid	LC-qTOF	Untargeted
Tryptophyl-Tryptophan	LC-qTOF	Untargeted
Tyrosol	LC-qTOF	Untargeted
Tyrosol	LC-qTOF	Untargeted
Undecanoyl-carnitine/Glutaconyl-carnitine/iso-undecanoyl-carnitine	LC-qTOF	Untargeted
Urolithin A	LC-qTOF	Untargeted
Urolithin B	LC-qTOF	Untargeted
Urolithin C	LC-qTOF	Untargeted
Ursolic acid	LC-qTOF	Untargeted
Valyl-Histidine	LC-qTOF	Untargeted
Valyl-Tryptophan	LC-qTOF	Untargeted

Supplementary Table S2: List of plasma metabolites analyzed

Name	Platform
11.13-Eicosadienoicacid	LC-qTOF
12.13-DiHOME(9)	LC-qTOF
12-HETE	LC-qTOF
15-HETE	LC-qTOF
1methylhistidine	LC-QqQ
20-HETE	LC-qTOF
2-hydroxyglutaricacid	LC-qTOF
3-Hydroxybutyricacid	LC-qTOF
3Methylhistidine	LC-QqQ
3-Phosphoglycericacid	LC-qTOF
5-Aminolevulinic acid	LC-QqQ
5-HETE	LC-qTOF
9.10-DiHOME(12)	LC-qTOF
9.10-EpOME(12)	LC-qTOF
9.12.13-TriHOME(10)	LC-qTOF
9-HODE/13-HODE	LC-qTOF
9-OxoODE	LC-qTOF
ABA	LC-QqQ
Adrenicacid	LC-qTOF
a-ketoglutaricacid	LC-qTOF
Alanine	LC-QqQ
androsteronesulfate-iso1	LC-qTOF
androsteronesulfate-iso2	LC-qTOF
androsteronesulfate-iso3	LC-qTOF
androsteronesulfate-iso4	LC-qTOF
androsteronesulfate-iso5	LC-qTOF
Arabitol	LC-qTOF
Arachidonicacid	LC-qTOF
Arginine	LC-QqQ
Asparagine	LC-QqQ
Asparticacid	LC-QqQ
a-tocopherol	LC-qTOF
BAIBA	LC-QqQ
bAlanine	LC-QqQ
Behenic acid (C22:0)	LC-qTOF
Betaine	LC-QqQ
C0	LC-QqQ
C10:0	LC-QqQ
C10:1	LC-QqQ

C12:0	LC-QqQ
C12:0-OH-a	LC-QqQ
C12:0-OH-b	LC-QqQ
C12:1	LC-QqQ
C14:0	LC-QqQ
C14:0-OH	LC-QqQ
C14:1	LC-QqQ
C14:2	LC-QqQ
C16:0	LC-QqQ
C16:0-OH	LC-QqQ
C16:1	LC-QqQ
C18:0	LC-QqQ
C18:1	LC-QqQ
C18:2	LC-QqQ
C2:0	LC-QqQ
C3:0	LC-QqQ
C4:0	LC-QqQ
C4:0-2M	LC-QqQ
C40-iso	LC-QqQ
C5:0	LC-QqQ
C5:1	LC-QqQ
C5-DC	LC-QqQ
C5-M-DC	LC-QqQ
C5-OH	LC-QqQ
C6:0	LC-QqQ
C8:0	LC-QqQ
C8:1	LC-QqQ
Chenodeoxycholic acid	LC-qTOF
ChoE(16:0)	LC-qTOF
ChoE(16:1)	LC-qTOF
ChoE(17:0)	LC-qTOF
ChoE(18:0)	LC-qTOF
ChoE(18:1)	LC-qTOF
ChoE(18:2)	LC-qTOF
ChoE(18:3)	LC-qTOF
ChoE(20:2)	LC-qTOF
ChoE(20:3)	LC-qTOF
ChoE(20:4)	LC-qTOF
ChoE(20:5)	LC-qTOF
ChoE(22:4)	LC-qTOF
ChoE(22:5)	LC-qTOF
ChoE(22:6)	LC-qTOF
Cholicacid	LC-qTOF

Choline	LC-QqQ
cis-10 heptadecenoicacid	LC-qTOF
cis-palmitoleicacid(palmitoleic)	LC-qTOF
Citricacid	LC-qTOF
Citrulline	LC-QqQ
Cortisol	LC-qTOF
Cortisone	LC-qTOF
Cystathione	LC-QqQ
Cystine	LC-QqQ
Deoxycholicacid	LC-qTOF
Deoxycholicacid-iso1	LC-qTOF
Deoxycholicacid-iso2	LC-qTOF
DG34:1	LC-qTOF
DG34:2	LC-qTOF
DG34:3	LC-qTOF
DG36:0	LC-qTOF
DG36:1	LC-qTOF
DG36:2	LC-qTOF
DG36:3	LC-qTOF
DG36:4	LC-qTOF
DHA	LC-qTOF
DHEAS	LC-qTOF
DiHODE	LC-qTOF
dihomo-y-linolenic acid-iso1	LC-qTOF
dihomo-y-linolenic acid-iso2	LC-qTOF
dihomo-y-linolenic acid-iso3	LC-qTOF
dihomo-y-linolenicacid	LC-qTOF
Dodecanoic acid (C12:0)	LC-qTOF
Eicosenoicacid	LC-qTOF
EPA	LC-qTOF
EpOME-iso2	LC-qTOF
EpOME-iso3	LC-qTOF
epoxy-stearicacid-iso1	LC-qTOF
epoxy-stearicacid-iso2	LC-qTOF
Ethanolamine	LC-QqQ
Fumaricacid	LC-qTOF
GABA	LC-QqQ
Glutamicacid	LC-QqQ
Glutamine	LC-QqQ
Glycericacid	LC-qTOF
Glycerol-1-phosphate	LC-qTOF
Glycine	LC-QqQ
Glycochenodeoxycholicacid	LC-qTOF

Glycocholic acid	LC-qTOF
Glycocholic acid-iso1	LC-qTOF
Glycodeoxycholicacid	LC-qTOF
Glycolicacid	LC-qTOF
Glycoursodeoxycholicacid	LC-qTOF
Heptadecanoic acid (C17:0)	LC-qTOF
Histidine	LC-QqQ
HODE-iso1	LC-qTOF
HpEPE	LC-qTOF
HpODE	LC-qTOF
Hydroxylysine	LC-QqQ
Hydroxyprolinetrans	LC-QqQ
Isoleucine	LC-QqQ
Kynurenine	LC-QqQ
Lacticacid	LC-qTOF
Leucine	LC-QqQ
Lignocericacid(C24:0)	LC-qTOF
Linoleicacid	LC-qTOF
Linolenicacid-iso1	LC-qTOF
Linolenicacid-iso2	LC-qTOF
Lithocholicacid	LC-qTOF
LPC14:0	GC-qTOF
LPC14:0-sn2	LC-qTOF
LPC15:0	LC-qTOF
LPC15:0-sn2	LC-qTOF
LPC16:0	LC-qTOF
LPC16:0e	LC-qTOF
LPC16:0-sn2	LC-qTOF
LPC16:1	LC-qTOF
LPC16:1e	LC-qTOF
LPC16:1-sn2	LC-qTOF
LPC17:0	LC-qTOF
LPC17:0-sn2	LC-qTOF
LPC17:1-sn1	LC-qTOF
LPC17:1-sn2	LC-qTOF
LPC18:0	LC-qTOF
LPC18:0e	LC-qTOF
LPC18:0-sn2	LC-qTOF
LPC18:1	LC-qTOF
LPC18:1-sn2	LC-qTOF
LPC18:2	LC-qTOF
LPC18:2-sn2	LC-qTOF
LPC18:3-sn1	LC-qTOF

LPC18:3-sn2	LC-qTOF
LPC19:0-sn1	LC-qTOF
LPC19:0-sn2	LC-qTOF
LPC19:1-sn1	LC-qTOF
LPC20:0	LC-qTOF
LPC20:0-sn2	LC-qTOF
LPC20:1	LC-qTOF
LPC20:1-sn2	LC-qTOF
LPC20:2	LC-qTOF
LPC20:2-sn2	LC-qTOF
LPC20:3	LC-qTOF
LPC20:3-sn2	LC-qTOF
LPC20:4	LC-qTOF
LPC20:4-sn2	LC-qTOF
LPC20:5-sn1	LC-qTOF
LPC20:5-sn2	LC-qTOF
LPC22:3-sn1	LC-qTOF
LPC22:3-sn2	LC-qTOF
LPC22:4-sn1	LC-qTOF
LPC22:4-sn2	LC-qTOF
LPC22:5-sn1	LC-qTOF
LPC22:6	LC-qTOF
LPC22:6-sn2	LC-qTOF
LPE14:0-sn1	LC-qTOF
LPE14:0-sn2	LC-qTOF
LPE15:0-sn1	LC-qTOF
LPE15:0-sn2	LC-qTOF
LPE16:0-sn1	LC-qTOF
LPE16:0-sn2	LC-qTOF
LPE16:1-sn1	LC-qTOF
LPE16:1-sn2	LC-qTOF
LPE17:0-sn1	LC-qTOF
LPE17:1-sn1	LC-qTOF
LPE17:1-sn2	LC-qTOF
LPE18:0-sn1	LC-qTOF
LPE18:0-sn2	LC-qTOF
LPE18:1-sn1	LC-qTOF
LPE18:1-sn2	LC-qTOF
LPE18:2-sn1	LC-qTOF
LPE18:2-sn2	LC-qTOF
LPE18:3-sn1	LC-qTOF
LPE18:3-sn2	LC-qTOF
LPE20:1-sn1	LC-qTOF

LPE20:1-sn2	LC-qTOF
LPE20:2-sn1	LC-qTOF
LPE20:3-sn1	LC-qTOF
LPE20:3-sn2	LC-qTOF
LPE20:4-sn1	LC-qTOF
LPE20:4-sn2	LC-qTOF
LPE20:5-sn1	LC-qTOF
LPE20:5-sn2	LC-qTOF
LPE22:4-sn1	LC-qTOF
LPE22:4-sn2	LC-qTOF
LPE22:5-sn1	LC-qTOF
LPE22:5-sn2	LC-qTOF
LPE22:6-sn1	LC-qTOF
LPE22:6-sn2	LC-qTOF
LPI14:0	LC-qTOF
LPI16:0	LC-qTOF
LPI16:1	LC-qTOF
LPI17:0	LC-qTOF
LPI18:0	LC-qTOF
LPI18:1	LC-qTOF
LPI18:2	LC-qTOF
LPI20:3	LC-qTOF
LPI20:4	LC-qTOF
LPI20:5	LC-qTOF
LPI22:4	LC-qTOF
LPI22:6	LC-qTOF
Lysine	LC-QqQ
Malicacid	LC-qTOF
Methionine	LC-QqQ
Myristicacid	GC-qTOF
Nervonic acid (C24:1 [cis-15])	LC-qTOF
N-Linoleoylethanolamine	GC-qTOF
N-oleoylethanolamine	GC-qTOF
Nonadecenoicacid	GC-qTOF
N-palmitoyl ethanolamine	GC-qTOF
N-stearoyl-ethanolamine	GC-qTOF
Oleicacid	GC-qTOF
Ornithine	LC-QqQ
OxoODE-iso1	GC-qTOF
OxoODE-iso2	GC-qTOF
Palmiticacid	GC-qTOF
PC30:0	LC-qTOF
PC31:0	LC-qTOF

PC32:0	LC-qTOF
PC32:1	LC-qTOF
PC32:1e	LC-qTOF
PC32:2	LC-qTOF
PC33:1	LC-qTOF
PC33:2	LC-qTOF
PC34:0	LC-qTOF
PC34:1	LC-qTOF
PC34:1e	LC-qTOF
PC34:2	LC-qTOF
PC34:2e	LC-qTOF
PC34:3	LC-qTOF
PC34:4	LC-qTOF
PC35:1	LC-qTOF
PC36:1	LC-qTOF
PC36:2	LC-qTOF
PC36:2e	LC-qTOF
PC36:3	LC-qTOF
PC36:4	LC-qTOF
PC36:4e	LC-qTOF
PC36:5	LC-qTOF
PC36:5e	LC-qTOF
PC38:2	LC-qTOF
PC38:3	LC-qTOF
PC38:4	LC-qTOF
PC38:4e	LC-qTOF
PC38:5	LC-qTOF
PC38:5e	LC-qTOF
PC38:6	LC-qTOF
PC40:4	LC-qTOF
PC40:4e	LC-qTOF
PC40:5	LC-qTOF
PC40:5e	LC-qTOF
PC40:6	LC-qTOF
PC42:5e	LC-qTOF
PE36:5e	LC-qTOF
PE38:5e	LC-qTOF
Pentadecanoicacid	GC-qTOF
Phenylalanine	LC-QqQ
Phosphoethanolamine	LC-QqQ
Pregnenolonesulfate	GC-qTOF
Proline	LC-QqQ
Pyruvicacid	LC-qTOF

Sarcosine	LC-QqQ
SDA	GC-qTOF
SDA-iso1	GC-qTOF
Serine	LC-QqQ
Serotonina	LC-QqQ
SM32:0	LC-qTOF
SM32:1	LC-qTOF
SM32:2	LC-qTOF
SM33:1	LC-qTOF
SM34:1	LC-qTOF
SM34:2	LC-qTOF
SM36:0	LC-qTOF
SM36:1	LC-qTOF
SM36:2	LC-qTOF
SM38:1	LC-qTOF
SM38:2	LC-qTOF
SM39:1	LC-qTOF
SM40:1	LC-qTOF
SM40:2	LC-qTOF
SM41:1	LC-qTOF
SM41:2	LC-qTOF
SM42:1	LC-qTOF
SM42:2	LC-qTOF
SM42:3	LC-qTOF
SM43:1	LC-qTOF
SM43:2	LC-qTOF
Sphinganine-1-P	GC-qTOF
Sphingosine-1-P	GC-qTOF
Stearicacid	GC-qTOF
Succinicacid	LC-qTOF
Taurine	LC-QqQ
Taurochenodeoxycholicacid	GC-qTOF
Taurocholicacid	GC-qTOF
Taurodeoxycholicacid	GC-qTOF
Taurolithocholicacid	GC-qTOF
Testosterone	GC-qTOF
TG46:0	LC-qTOF
TG46:1	LC-qTOF
TG46:2	LC-qTOF
TG47:0	LC-qTOF
TG47:1	LC-qTOF
TG48:0	LC-qTOF
TG48:1	LC-qTOF

TG48:2	LC-qTOF
TG48:3	LC-qTOF
TG50:0	LC-qTOF
TG50:1	LC-qTOF
TG50:2	LC-qTOF
TG50:3	LC-qTOF
TG50:4	LC-qTOF
TG51:2	LC-qTOF
TG52:1	LC-qTOF
TG52:2	LC-qTOF
TG52:3	LC-qTOF
TG52:4	LC-qTOF
TG52:5	LC-qTOF
TG54:2	LC-qTOF
TG54:3	LC-qTOF
TG54:4	LC-qTOF
TG54:5	LC-qTOF
TG54:6	LC-qTOF
TG54:7	LC-qTOF
TG56:5	LC-qTOF
TG56:6	LC-qTOF
TG56:7	LC-qTOF
TG58:8	LC-qTOF
Threonicacid	LC-qTOF
Threonine	LC-QqQ
TMA	LC-QqQ
TMAO	LC-QqQ
Tryptophan	LC-QqQ
Tyrosine	LC-QqQ
Ursodeoxycholic/Hyodeoxicholicacid	GC-qTOF
Valine	LC-QqQ
w3-DPA	GC-qTOF
w6-DPA	GC-qTOF

Supplementary Table S3: Summary statistics of the network analysis

**Number of nodes**	94
**Number of edges**	263
**Av.g number of neighbors**	6733
**Network diameter**	4
**Network radius**	2
**Characteristic path lenght**	2,041
**Network density**	0,232
**Network heterogeneity**	0,709
**Network centralization**	0,416
**Connected components**	5

Figure S1: Description of the connected components of the network

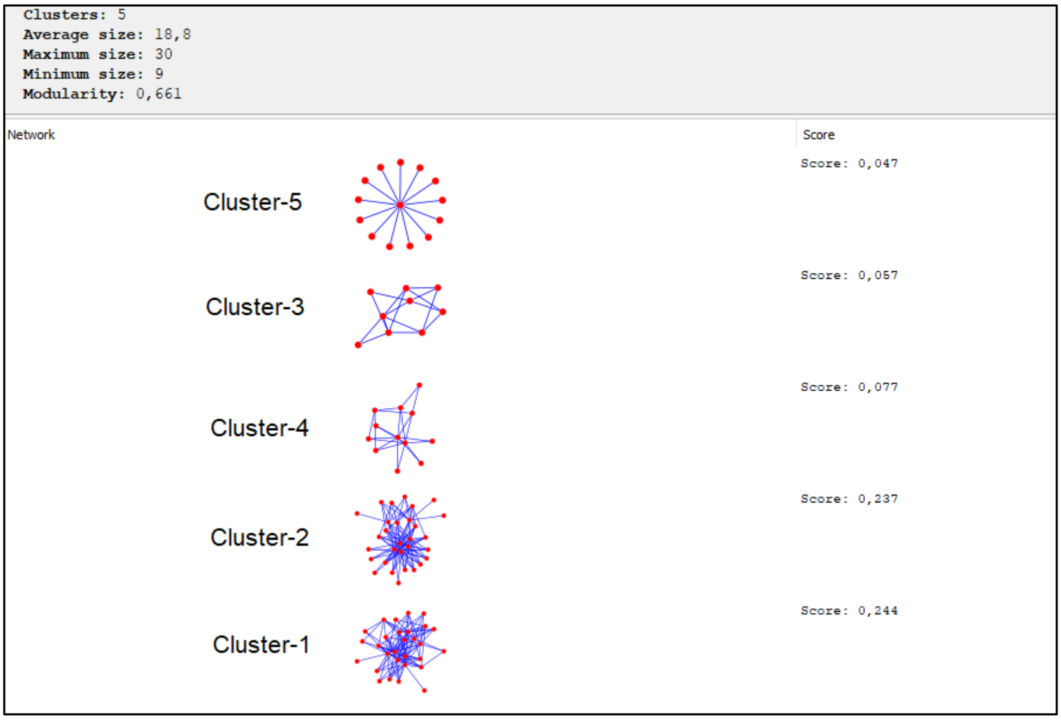

Figure S2: Heatmap of correlations from the network similarity matrix between selected plasma and fecal metabolites*

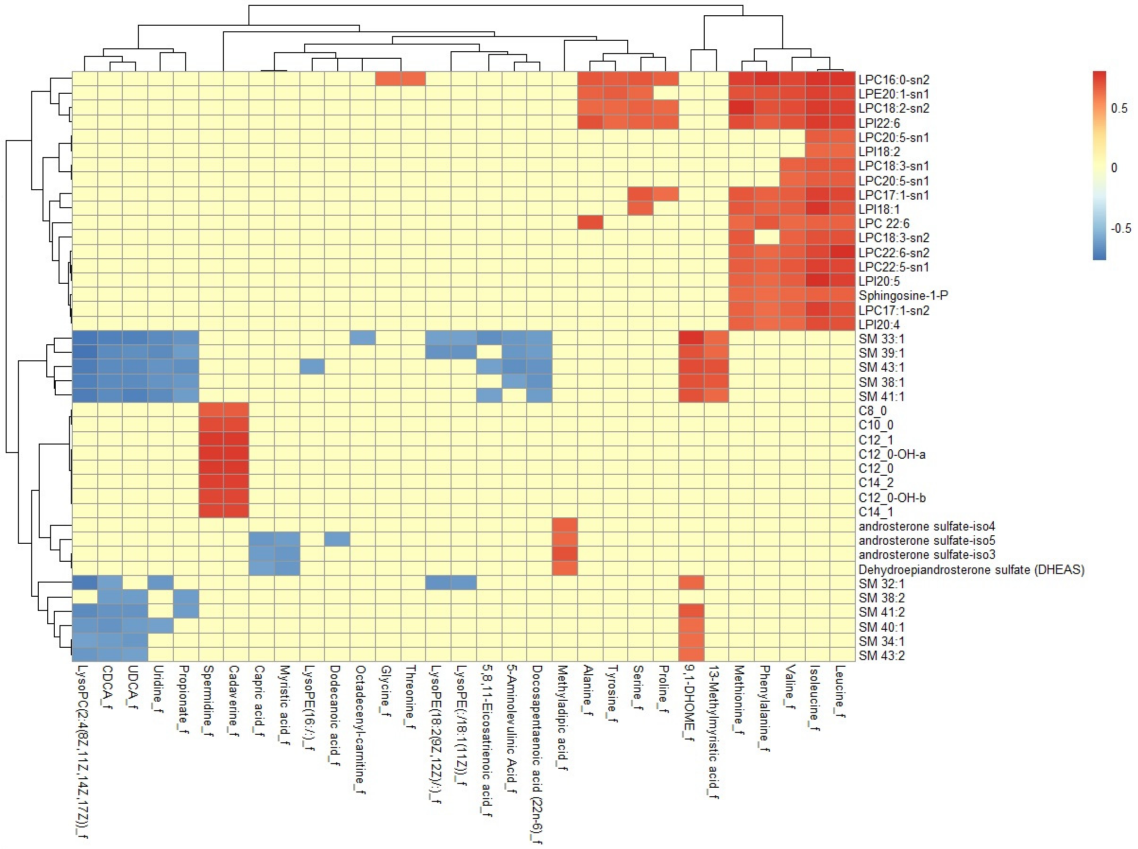

*_f stands for fecal metabolites

Figure S3: Heatmap of correlations from the network similarity matrix between selected plasma metabolites and microbial genera

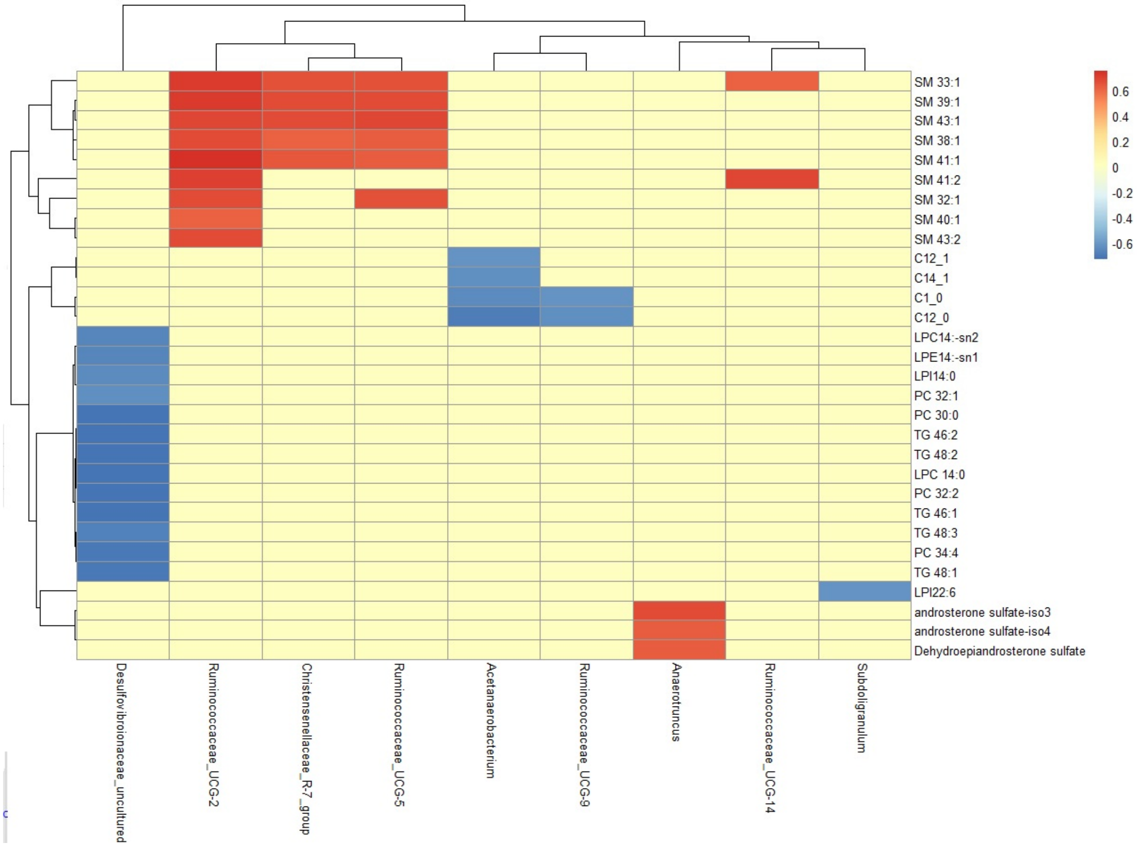

Figure S4: Heatmap of correlations from the network similarity matrix between selected fecal metabolites and microbial genera*

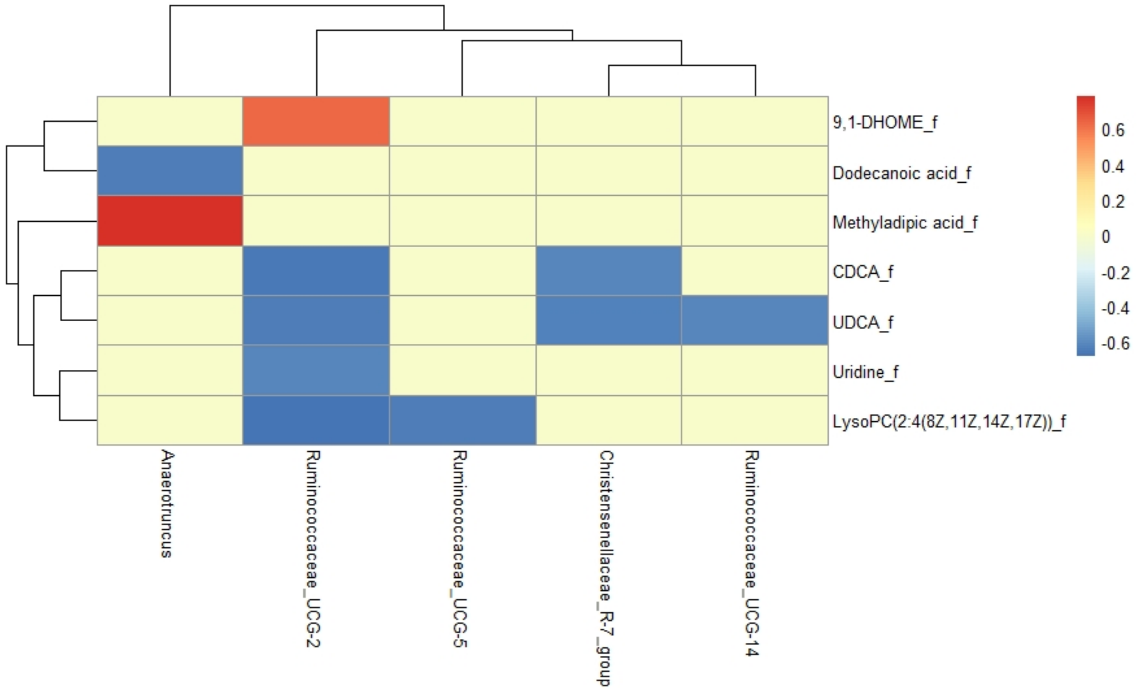

*_f stands for fecal metabolites

References

[1] J. Wang, L. Zhou, H. Lei, et al. Simultaneous Quantification of Amino Metabolites in Multiple Metabolic Pathways Using Ultra-High Performance Liquid Chromatography with Tandem-mass Spectrometry. *Sci Rep*. Published online 2017 DOI:10.1038/s41598-017-01435-7.

[2] T.B.K.M.S.T.P. David F. Automated Sample Preparation for Profiling Fatty Acids in Blood and Plasma using the Agilent 7693 ALS. *Agilent*. Published online 2009.
